# Predicting in-season maize (*Zea mays* L.) yield potential using crop sensors and climatological data

**DOI:** 10.1038/s41598-020-68415-2

**Published:** 2020-07-10

**Authors:** Jagmandeep Dhillon, Lawrence Aula, Elizabeth Eickhoff, William Raun

**Affiliations:** 1 Department of Plant and Soil Sciences, Mississippi State University, 470 Dorman Hall, Starkville, MS 39762 United States; 2 Department of Plant and Soil Sciences, Oklahoma State University, 368 Agricultural Hall, Stillwater, OK 74078 United States

**Keywords:** Plant sciences, Environmental sciences

## Abstract

The environment randomly influences nitrogen (N) response, demand, and optimum N rates. Field experiments were conducted at Lake Carl Blackwell (LCB) and Efaw Agronomy Research Station (Efaw) from 2015 to 2018 in Oklahoma, USA. Fourteen site years of data were used from two different trials, namely Regional Corn (Regional) and Optimum N rate (Optimum N). Three algorithms developed by Oklahoma State University (OSU) to predict yield potential were tested on both trials. Furthermore, three new models for predicting potential yield using optical crop sensors and climatological data were developed for maize in rain-fed conditions. The models were trained/built using Regional and were then validated/tested on the Optimum N trial. Out of three models, one model was developed using all of the Regional trial (combined model), and the other two were prepared from each location LCB and Efaw model. Of the three current algorithms; one worked best at predicting final grain yield at LCB location only. The coefficient of determination R^2^ = 0.15 and 0.16 between actual grain yield and predicted grain yield was observed for Regional and Optimum N rate trials, respectively. The results further indicated that the new models were better at predicting final grain yield except for Efaw model (R^2^ = 0.04) when tested on optimum N trial. Grain yield prediction for the combined model had an R^2^ = 0.31. The best yield prediction was obtained at LCB with an R^2^ = 0.52. Including climatological data significantly improved the ability to predict final grain yield along with using mid-season sensor data.

## Introduction

The three most yield-limiting nutrients in cereal crop production are N, phosphorus (P), and potassium (K). Globally, N consumption has increased eight times^1^, P increased 3.5^2^ and K consumption has increased three times^3^, since the year 1961. Undoubtedly, N remains the most consumed nutrient worldwide.

A review in 1999 encompassing global N use and cereal crop production found that nitrogen use efficiency (NUE) in cereal crops was 33%^4^. A lot of research has been devoted to improving NUE in crops grown worldwide^4–6^. Lassaletta et al.^5^ noted that considering the present situation increasing N fertilization would not result in a yield increase. Alternatively, they suggested improvement of agronomic practices to be an efficient strategy. Raun and Johnson^4^ proposed various approaches through which NUE could be improved, including site-specific N management.

Historically, yield goals have been used for estimating preplant^7^ and in-season N rates^8^. However, their adequacy has been refuted in winter wheat^9^, and maize^10^. Rodriguez et al.^10^ deduced data-intensive fertilizer management as a more efficient N management strategy. Crop optical sensing has been effectively used to identify N deficiency in the field and determine in-season N fertilizer recommendation^11–17^.

The first and critical step for sensor-based site-specific nutrient management is in-season prediction of yield potential^3,8,17^, for which an algorithm is required^18,19^. The optical sensors use normalized difference vegetation indices (NDVI) from crop canopy reflectance of red and near-infrared wavebands to estimate chlorophyll content, crop N content, biomass, and yield predictions^16,20–22^. To improve yield estimates, Raun et al.^7^ introduced an in-season estimate of yield (INSEY), using NDVI divided by growing degree days where yield was possible (GDD > 0) in winter wheat. Maize algorithms for determining N recommendations utilizes the measurement of the crop grain yield potential at the time of sensing along with a response index (RI), a ratio of N-rich NDVI compared to a deficient N area^23^.

Numerous researchers have tried to improve the in-season estimate of yield by including other variables in conjunction with NDVI and GDD. Girma et al.^24^ combined chlorophyll content, plant height, and total N uptake along with NDVI as good predictors of final wheat grain yield. Walsh et al.^25^ included soil moisture at 0.05 m depth with NDVI to accurately predict wheat grain yield. Bushong et al.^8^ assimilated NDVI, days of potential growth (based on temperature and soil moisture), and a stress index (amount of plant-available water divided by the amount of water required to maintain yield) to accurately predict in season wheat yield estimates. In maize, a combined model using plant height along with NDVI was used to successfully predict by-plant corn grain yields at the V8 growth stage^21,26^. Sharma et al.^15^ included soil moisture in a polynomial model of yield prediction and concluded it to be better than exponential models. Machine learning (ML) offers another new possibility to predict N fertilizer recommendation in maize. The difference between ML and traditional statistics is the inclusion of more variables in machine learning^27^. Puntel et al.^28^ used soil depth, precipitation, heat stress, nitrate–N at planting, and residue amount, out of 54 variables used in the study, to predict economic optimum N rate.

Morris et al.^13^ found it difficult to accurately predict the amount of N needed by maize and inferred that current techniques are inadequate in N recommendations. In their review, they concluded that uncontrollable environmental factors and their interaction with other considerations such as N source, timing and placement, plant genetics, and soil characteristics need to be combined to make better N rate recommendations. Recently, Raun et al.^29^ suggested independent estimation of multiple random variables for mid-season algorithms due to erratic effect environment has on N demand and final grain yield.

The studied area in this work is in the Southern Great Plains (SGP). The SGP mainly comprises of Texas, Kansas, and Oklahoma, and this region frequently encounters periods of prolonged drought, erratic rainfall, and variable air temperatures^30^. The variability that is encountered in precipitation and temperature within SGP affects nutrient management for various crops. Consequently, the first objective of this study was to assess the efficiency of three current algorithms developed by Oklahoma State University (OSU) to predict yield potential. Furthermore, the second objective was to create three new models for predicting potential yield using optical crop sensors and climatological data for maize under rainfed conditions.

## Results

### Evaluation of current algorithms

**Figure 1:**
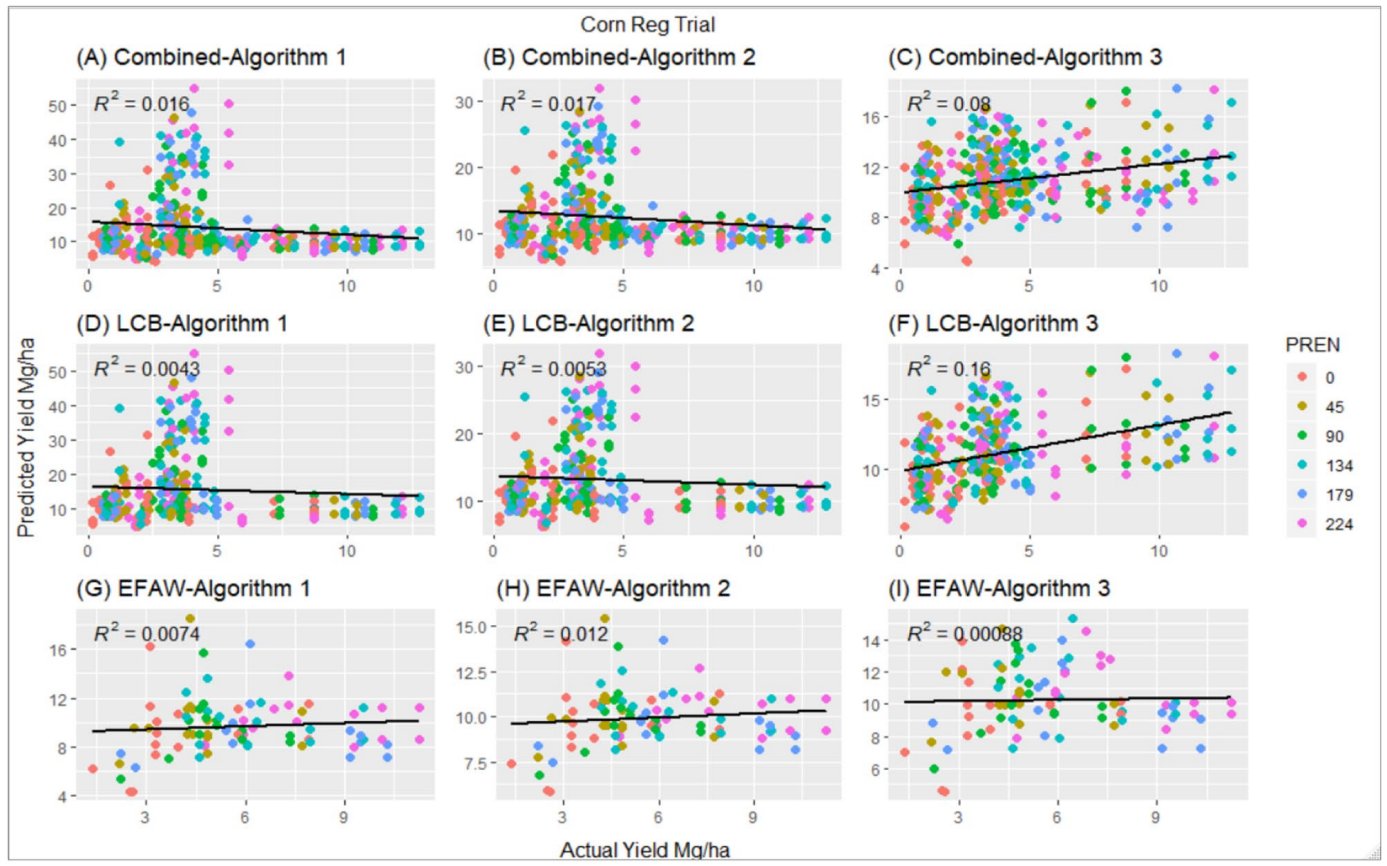
Correlation between actual yield and predicted yield using three current algorithms at different preplant N rates (PREN), Corn Regional trial, using combined data with algorithm 1 (**A**), using combined data with algorithm 2 (**B**), using combined data with algorithm 3 (**C**), using LCB data with algorithm 1 (**D**), using LCB data with algorithm 2 (**E**), using LCB data with algorithm 3 (**F**), using Efaw data with algorithm 1 (**G**), using Efaw data with algorithm 2 (**H**), using Efaw data with algorithm 3 (**I**).

All the three algorithms performed poorly when tested on combined data for both locations for the Regional trial (Fig. 1). There was a weak correlation among predicted and actual grain yield evident by the lower coefficient of determination (R^2^ < 0.08).

**Figure 2:**
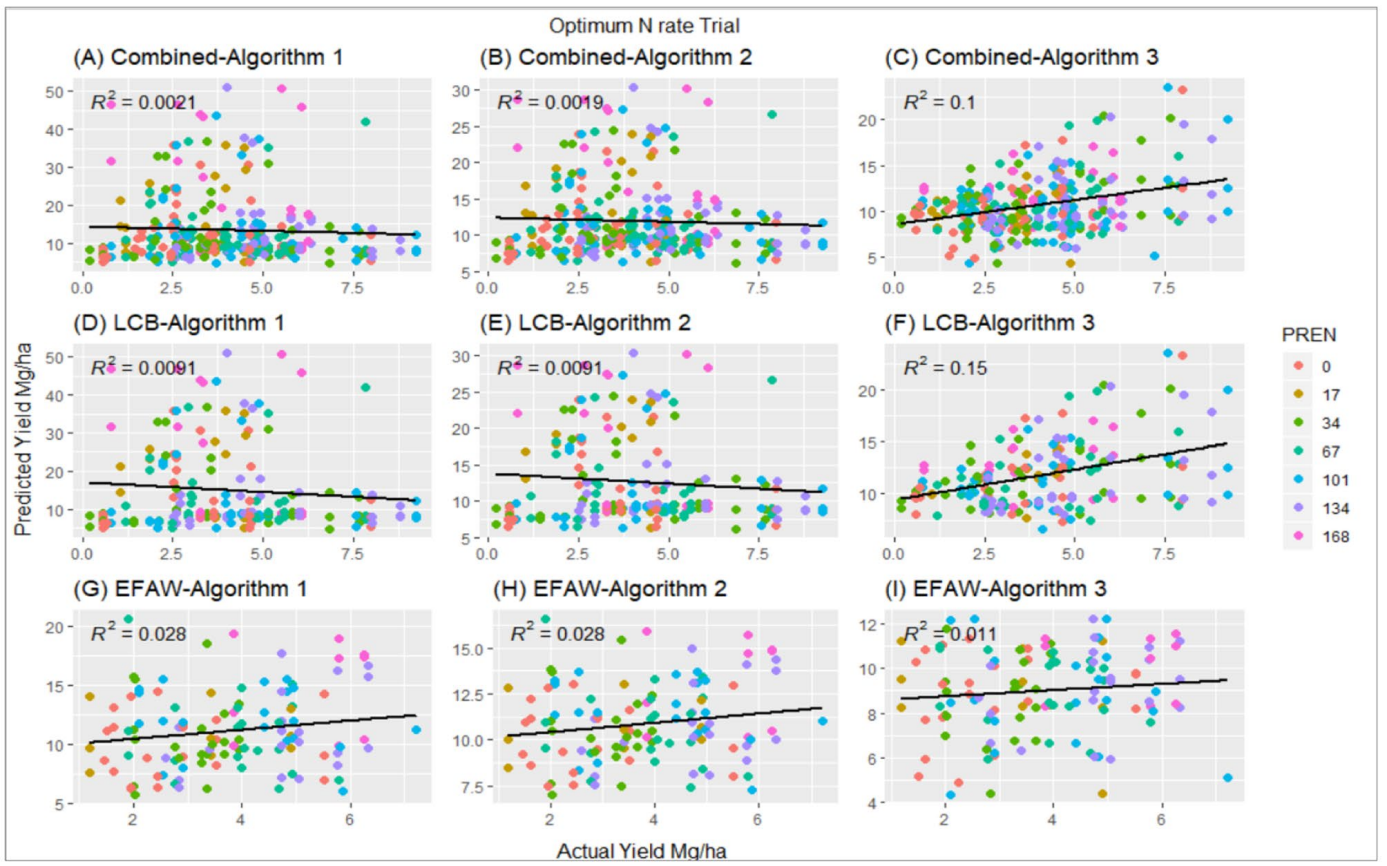
Correlation between actual yield and predicted yield using three current algorithms at different preplant N rates (PREN), Optimum N rate trial, using combined data with algorithm 1 (**A**), using combined data with algorithm 2 (**B**), using combined data with algorithm 3 (**C**), using LCB data with algorithm 1 (**D**), using LCB data with algorithm 2 (**E**), using LCB data with algorithm 3 (**F**), using Efaw data with algorithm 1 (**G**), using Efaw data with algorithm 2 (**H**), using Efaw data with algorithm 3 (**I**).

At LCB algorithms 1 and 2 were unable to predict yields. The predicted yield with algorithm 1 and 2, at higher PREN, resulted in the prediction of yields up to 50 Mg ha^−1^. However, algorithm 3 performed slightly better with an R^2^ = 0.16. Similar to combined sites, all the algorithms were unable to predict actual grain yield at Efaw. Similar to the Regional trial, all three algorithms performed poorly at predicting final grain yield in Optimum N trial (Fig. 2). Lower R^2^ were observed with all three algorithms for all the combined data. At LCB, algorithm 3 was able to explain 16% of the actual yield, where algorithm 1 and 2 explained only 0.5%. None of the algorithms were able to explain more than 1.2% of actual grain yield at Efaw location.

### New models

All three models built using Regional data are reported in Table 1. The first model was developed using all seven site years of Regional data and included 8 out of 66 explanatory variables included in this study. The Cp, AIC, BIC, and adjusted R^2^, all selected eight variables as an optimal model. Based on their importance, the first variable included in the model was PREN, followed by NDVI, and GDD. The next variable added was the average monthly soil temperature at 250 mm under sod for July (S25AVG-7). The last four variables included were fractional water indices at 250 mm for April (FWI25-4), June (FWI25-6), July (FWI25-7), and September (FWI25-9). This model could explain 83% of the final grain yield (Table 1).

**Table 1:** New regression models for predicting final grain yields.

Data	Model	Adjusted R^2^
Combined	− 2.751386e + 02 + 9.087027e−03 × PREN + 3.165433e + 00 × NDVI − 3.346925e−03 × GDD + 8.599644e + 00 × S25AVG.7 + 3.228292e + 01 × FWI25.4 + 7.775841e + 00 × FWI25.6 + 1.190534e + 01 × FWI25.7 + 6.451529e + 00 × FWI25.9	0.83
Lake Carl Blackwell	− 6.408236013 + 0.006625366 × PREN + 3.271162048 × NDVI − 0.003379189 × GDD- 0.019196793 × AprR + 0.331984242 × SAVG.8 + 9.377433891 × FWI25.9	0.85
Efaw	− 94.63931672 + 0.01725896 × PREN − 1.14674051 × BAVG.8 + 4.74565389 × S25AVG.7	0.67

The acronyms are explained in Table 4.

The second model trained using LCB Regional data included six variables as per Cp, AIC, BIC, and adjusted R^2^ (Table 1). This model included PREN, NDVI, GDD, and FWI25-9, similar to the combined model. Furthermore, it included average precipitation for April (AprR) and average monthly soil temperature 100 mm under sod (SAVG-8). This was the best model of the three new models with the ability to explain 85% of final grain yield.

The third model only included three variables and could only clarify 67% of the final yield and was built using Efaw Regional data (Table 1). The first variable included in this model was also PREN, followed by average monthly soil temperature 100 mm under bare soil for August (BAVG-8). The last variable included was S25AVG-7.

### New model validation

**Figure 3:**
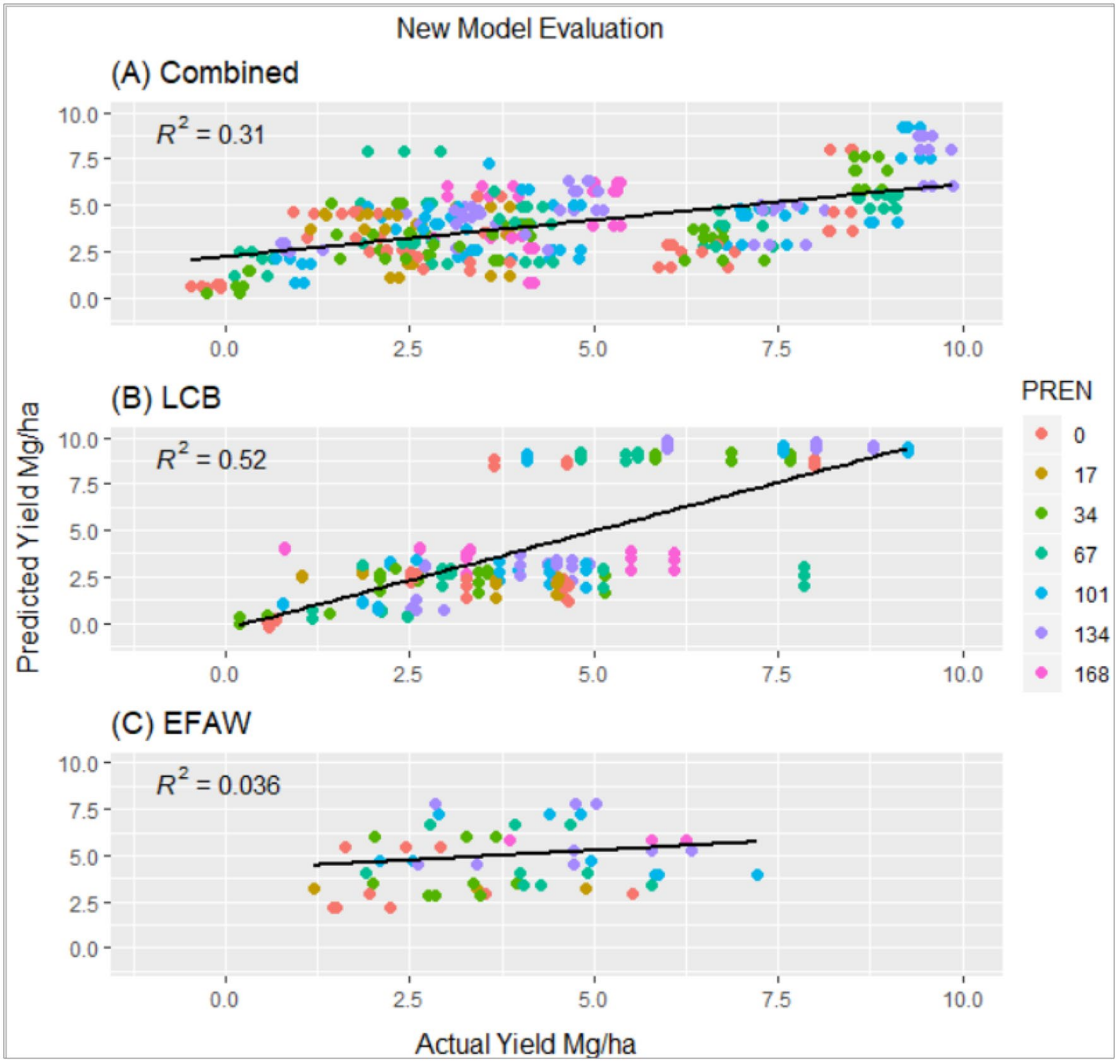
Validation of new models at different preplant N rates (PREN) on Optimum N rate trial, using combined data with the combined model (**A**), using LCB data with LCB model (**B**), using Efaw data with Efaw model (**C**).

The combined model performed better than current algorithms when tested on all site years of optimum N rate. The coefficient of determination (R^2^) between actual yield and model predicted yield was 0.31 (Fig. 3); a figure higher than that of any existing algorithms. The LCB model was best at predicting final grain yield with an R^2^ = 0.52. The Efaw model was unable to predict final grain yield and only explained 3.6% of the actual grain yield.

## Discussion

The INSEY approach could result in overestimation of potential yield^23,31^. This happened while testing the current algorithm 1 and 2. The high PREN rates resulted in higher NDVI values. Incidentally, the projected biomass produced per day was large with relatively lower GDD. Extreme changes in growing condition from sensing to harvesting would lower the actual yield creating a vast difference in real and predicted yields^31^. This could be avoided by supplementing INSEY with more risk aversion prediction models^31^. Furthermore, locally developed algorithms do not perform outside the region for which they were designed^32^. All three current OSU algorithms were built on data before 2010, and any inconsistency in newer data (environment, genetics, soil types, etc.) would affect their efficiency. Moreover, temporal restriction needs to be considered, necessitating consistent upgrading of these algorithms.

In this study, we employed supervised learning category of ML involving 66 explanatory variables for yield prediction. After removing highly correlated variables, only 15 variables remained, and those were used to build new models. Puntel et al.^28^ developed new models using ML for the central-west region of Argentina. We developed predictive models for yield potential by combining optical sensors and climatological data. In agreement with Puntel et al.^28^, our models use fewer inputs than a simulation models.

Furthermore, our models are significant in comparison to the other current methodologies and account for spatial and temporal variability, appropriate for application in precision agriculture. In-season N assessment using optical sensor is a promising approach over other traditional methods^13,14,23,33,34^. However, their precision depends on the accuracy of algorithms that convert crop reflectance to yield prediction and N recommendation^13^. Analogous to this, we tried to develop regional algorithms to closely mimic factors such as current crop variety, rainfall, soils, timing, form, and placement of N, and interactions of these factors to determine potential yield and recommend N rates regionally as highlighted by^13^.

Bushong et al.^8^ stressed proper validation of developed prediction models, to determine the accuracy of models to predict grain yield on an independent data set. They further highlighted the scarcity of work that validates yield prediction models. Numerous models explain the goodness of fit of the data used to develop the model without validation with an independent data set^15,17,21,23–25,31,35^. Our new models were independently validated, and where two out of three models performed well with a prediction accuracy of 31% and 52%.

## Conclusion

In this work, an attempt to derive data-driven recommendations using NDVI and weather information currently collected by Mesonet weather stations was made. These regional models have the efficiency to explain final grain yield with 67%, 83%, and 85% accuracy, which is a crucial step for making N recommendations. When tested on an independent data set these models accurately predicted 31% and 52% final grain yield. However, in the future, it is essential to keep these models apprised with newer data.

## Methods

### Site description

The corn regional experiment (Regional) was set up in a randomized complete block design with 12 treatments replicated three times. This trial was initiated to update the algorithms used for yield prediction and subsequent N rate recommendation. A total of 7 site-years of data were included in this study. Data from Lake Carl Blackwell (LCB) from 2015 to 2018 were used. Three-years of data from 2016 to 2018 from Efaw Agronomy Research Station (Efaw) were also included. This experiment was further used to train/build three new models for yield prediction.

Similarly, a randomized complete block experimental design with three replications at all sites with fourteen treatments was used for the Optimum N rate experiment (Optimum N). This study was initiated to predict the optimum preplant N rates for the region. Similar to the Regional trial, a total of 7 site-years of data were included in this study. Data from four years at LCB and three at Efaw were analyzed. Both experiments were fertilized to a 100% level based on P and K test following regional fertilizer recommendations^36^, to ensure N was the only limiting nutrient. This trial was further used to validate/test new models built using regional corn data. Soil descriptions for each site are reported in Table 2.

**Table 2:** Description of soil series at Efaw and Lake Carl Blackwell, OK.

Location	Soil series
Efaw	Ashport silty clay loam (fine-silty, mixed, superactive, thermic Fluventic Haplustolls)
Lake Carl Blackwell	Port silt loam (fine-silty, mixed, thermic Cumulic Haplustolls)

For all sites, only treatments with no N fertilizer or preplant N (PREN) fertilizer applications were included in this study. Treatments with mid-season N application were excluded. A total of six treatments from Regional, and seven treatments from Optimum N trial were included. The treatments and N rates included for each trial location are listed in Table 3.

**Table 3:** Nitrogen fertilizer application rate used for model testing and training of this study.

Trial (replication)	Location	N		Plot size
		Source	Preplant Rate (kg N ha^−1^)	
Corn regional (3)	LCB	UAN (28–0–0)	0, 45, 90, 134, 179, 224	3.0 m × 6.1 m
	Efaw	UAN (28–0–0)	0, 45, 90, 134, 179, 224	3.0 m × 6.1 m
Optimum N trial (3)	LCB	UAN (28–0–0)	0, 17, 34, 67, 101, 134, 168	3.0 m × 6.1 m
	Efaw	UAN (28–0–0)	0, 17, 34, 67, 101, 134, 168	3.0 m × 6.1 m

### Field data collection

Throughout the growing season, NDVI data were collected using a hand-held Greenseeker sensor from V4 to V8 growth stages. The GreenSeeker sensor measured NDVI by utilizing the Eq. (1):

1 $${\text{NDVI }} = \frac{\rho NIR - \rho RED}{{\rho NIR + \rho RED}}$$

where near-infrared (NIR) was measured at 780 nm and red at 650 nm^37^. A self-propelled Massey Ferguson 8XP Combine (AGCO Corp., Duluth, GA, USA) equipped with harvest master (Juniper Systems Inc., Logan, UT, USA) automated weighing systems were used for harvesting the center two of four-row plots. The final yield was adjusted to 15.5% moisture content.

The weather data was taken from the Oklahoma Mesonet climate monitoring station^38^. Sensor data was normalized to calculate a fractional water index (FWI), which is a unitless value ranging from 0.00 for dry soils to 1.00 for the wet/saturated soils^39^. The list of all the other explanatory variables included in this study is listed in Table 4.

**Table 4:** Description of all the explanatory variables used in this study.

Acronym	Explanation	Unit
MarR, AprR, MayR, JunR, JulR, AugR, SepR	Average monthly rainfall from March to September	mm
TAVG-3,TAVG-4,TAVG-5,TAVG-6,TAVG-7,TAVG-8,TAVG-9	Average monthly air temperature from March to September	Degrees celsius (C)
SAVG-3,SAVG-4,SAVG-5,SAVG-6,SAVG-7,SAVG-8,SAVG-9	Average monthly soil temperature 100 mm under sod from March to September	C
BAVG-3,BAVG-4,BAVG-5,BAVG-6,BAVG-7,BAVG-8,BAVG-9	Average monthly soil temperature 100 mm under bare soil from March to September	C
S5AVG-3,S5AVG-4,S5AVG-5,S5AVG-6,S5AVG-7,S5AVG-8,S5AVG-9	Average monthly soil temperature 50 mm under sod from March to September	C
S25AVG-3,S25AVG-4,S25AVG-5,S25AVG-6,S25AVG-7,S25AVG-8,S25AVG-9	Average monthly soil temperature 250 mm under sod from March to September	C
S60AV-3,S60AV-4,S60AV-5,S60AV-6,S60AV-7,S60AV-8,S60AV-9	Average monthly soil temperature 600 mm under sod from March to September	C
FWI05-3,FWI05-4,FWI05-5,FWI05-6,FWI05-7,FWI05-8,FWI05-9	Monthly Fractional water index at 50 mm for each month from March to September	Unitless
FWI25-3,FWI25-4,FWI25-5,FWI25-6,FWI25-7,FWI25-8,FWI25-9	Fractional water index at 250 mm for each month from March to September	Unitless

### Data analysis

#### Evaluation of a current model for yield prediction

Three algorithms from (https://www.nue.okstate.edu/Yield_Potential.htm) were evaluated to predict yield potential. All these algorithms were made using the methodology described by Raun et al.^23^. A linear regression analysis among predicted and actual yield was conducted to check the efficiency of these algorithms. This was done on both regional and optimum N rate data with the combined data and then for each location separately.

The following algorithms were used:

1. YP0 = 2,592 × (EXP(NDVI/Sum of GDD × 1775.6); RI Harvest = 1.64(RI-NDVI) − 0.5287
2. YP0 = 1,287 × (EXP(NDVI/Sum of GDD × 2,655); RI Harvest = 1.64(RI NDVI)  − 0.5287
3. YP0 = (CoefA × EXP (CoefB × NDVI)); RI Harvest = 1.64(RI-NDVI)  − 0.5287
  CoefA = 641.4158203057011 + 4207.148880805758/(1.0 + EXP(− (x − 897.0822110817790)/(− 32.78891349907328)))
  CoefB = 1.46923333343772 + 1.8752166665474/(1 + EXP(− (x − 912.164821648278)/2.66689327528455))
  (x = cumulative GDD)

#### Training new models for yield prediction

New models for yield prediction were trained using data from the regional trial and climatological data obtained from the Oklahoma Mesonet climate monitoring station. A total of 66 explanatory variables were included for a multiple linear regression model comprised of preplant N rate, NDVI, Growing degree day heat units (GDD) along with weather variables listed in Table 4. The NDVI data were used for the growth stages from V4 to V8, and due to inconsistency in data collection, the new models were not separated for each growth stage. The climatological variables included encompassed the months of March to September.

Following steps were taken to generate the new models:

1. A Pearson correlation test was conducted for all the explanatory variables to remove highly correlated variables. The R statistical package “Hmisc” was used to calculate correlation coefficients and p-values. For plotting correlograms R package, “corrplot” was used.
2. Highly correlated variables were excluded from subsequent regression analysis; a reduced number of variables were used in the next step.
3. Best subset selection was used to identify a subset of variables. This reduced set was then fitted using least squares to predict yield. The single best model was then selected using cross-validation prediction error (Cp), Akaike information criterion (AIC), Bayesian information BIC, and adjusted R^2^. All these approaches estimate test error by adjusting the training error to account for overfitting due to the bias^40^. The branch and bound algorithm within “leaps” package of R statistical software^41^ were used for best sub selection.
4. Three different linear regression models were developed. The first one using all of the data from regional termed as the combined model and the other two for each location, LCB, and Efaw, respectively.

#### Testing new model for yield prediction

The new models were tested on Optimum N trials. The three new models were tested by conducting a correlation between model predicted yields and actual yields. The combined model was tested on all of the data from Optimum N experiment. Only LCB data from Optimum N trial was used to validate the LCB model, and similarly, Efaw Optimum N data was used to check the Efaw model.
